# Readministration of gefitinib in a responder after treatment discontinuation due to gefinitib-related interstitial lung disease: a case report

**DOI:** 10.1186/1752-1947-1-138

**Published:** 2007-11-17

**Authors:** Kazuya Takamochi, Kazuya Suzuki, Abul Hasan Muhammad Bashar, Kiyoshige Yajima, Takahiro Mochizuki, Toru Itaya, Kazuhito Funai

**Affiliations:** 1 First Department of Surgery, Hamamatsu University School of Medicine, 1-20-1 Handayama, Hamamatsu, 435-3192 Japan

## Abstract

**Introduction:**

Gefitinib is a new molecular-targeted agent for the treatment of patients with advanced non-small cell lung cancer that fail to respond to conventional chemotherapy. Gefitinib is considered to be well tolerated and less toxic compared with conventional cytotoxic drugs. However, interstitial lung disease (ILD) has been reported as a serious adverse effect. The precise management of a gefitinib responder having severe adverse events remains unknown.

**Case Presentation:**

We report the case of gefitinib readministration in a patient with lung adenocarcinoma who had once responded but in whom treatment had to be discontinued owing to gefinitib-related ILD. A dramatic response was achieved both at the time of initial treatment (250 mg/day) and at readministration of gefitinib (125 mg/day). The effectiveness of gefitinib therapy in our patient could be explained in part by the presence of an activating mutation of epidermal growth factor receptor (*EGFR*) gene, L858R in exon 21, which was identified in the primary tumor.

**Conclusion:**

A reduced dose of gefitinib might be sufficient for patients having tumors with *EGFR *gene mutations, and that the currently approved dose may be excessively potent in some of these patients, thus resulting in the onset of adverse events.

## Introduction

Gefitinib is a new molecular-targeted agent for the treatment of patients with advanced non-small cell lung cancer that fail to respond to conventional chemotherapy. Early clinical trial data demonstrated that gefitinib was well tolerated and was less toxic compared with conventional cytotoxic drugs[1,2]. The most common adverse events were skin rash and diarrhea, which are reversible with discontinuation of treatment. However, gefitinib-related interstitial lung disease (ILD) has been reported as a serious adverse effect of gefitinib therapy [3,4]. The largest retrospective study conducted by the West Japan Thoracic Oncology Group (WJTOG) showed an overall prevalence of 3.5% and a mortality of 1.6% [4] Although the precise mechanism of gefitinib-related ILD remains unknown, the WJTOG study showed in a multivariate analysis that male sex, a history of smoking, and the coexistence of interstitial pneumonia were all significant risk factors.

We herein report a thought-provoking case of readministration of gefitinib in a patient with lung adenocarcinoma who had previously responded and developed gefinitib-related ILD. Gefitinib readministration with 50% dose was successful in managing disease progression.

## Case presentation

A 56-year-old male was referred to our hospital because of a lung nodule detected on population-based radiological screening. He was a current smoker with a smoking index of 15 pack-years. A chest computed tomography (CT) scan demonstrated a15 mm solid-density nodule with pleural indentation in the left lower lobe. Mediastinal lymph nodes were not swollen in the mediastinal setting images (clinical T1N0M0, stage IA). He underwent left lower lobectomy and systematic lymphadenectomy. He was diagnosed as having lung adenocarcinoma with multiple mediastinal lymph node metastases and solitary pulmonary metastasis (pathological T4N2M0, stage IIIB). He received two courses of postoperative adjuvant chemotherapy (carboplatin/paclitaxel).

**Figure 1:**
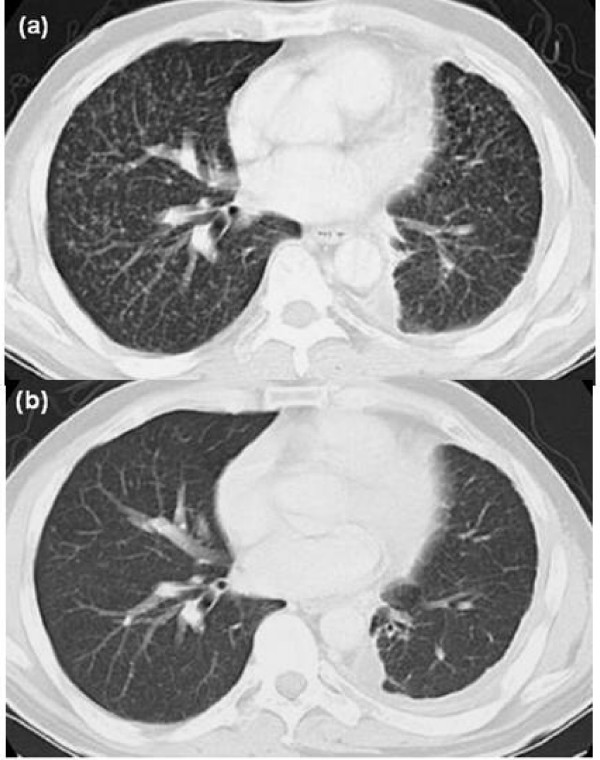
Radiological evaluation of response to initial treatment with gefitinib on CT cans. (a) before treatment, (b) after treatment.

Multiple bone metastases developed 10 months after the operation. He received two courses of chemotherapy (cisplatin/docetaxel) and palliative irradiation therapy. Subsequently, at 17 months after the operation, a follow-up CT scan indicated miliary pulmonary metastases with lymphangitis carcinomatosa throughout both lungs and mediastinal lymphadenopathy (Figure 1a). ILD was not evident on a chest CT scan. No respiratory symptoms were noted. Multiple brain metastases were simultaneously detected on brain magnetic resonance imaging. The oral administration of gefitinib 250 mg/day and whole brain irradiation therapy (total 30 Gy/12 fr) were initiated. A rapid improvement in multiple pulmonary metastases was observed 14 days after the administration of gefitinib (Figure 1b). Brain metastatic lesions showed a mild regression.

**Figure 2:**
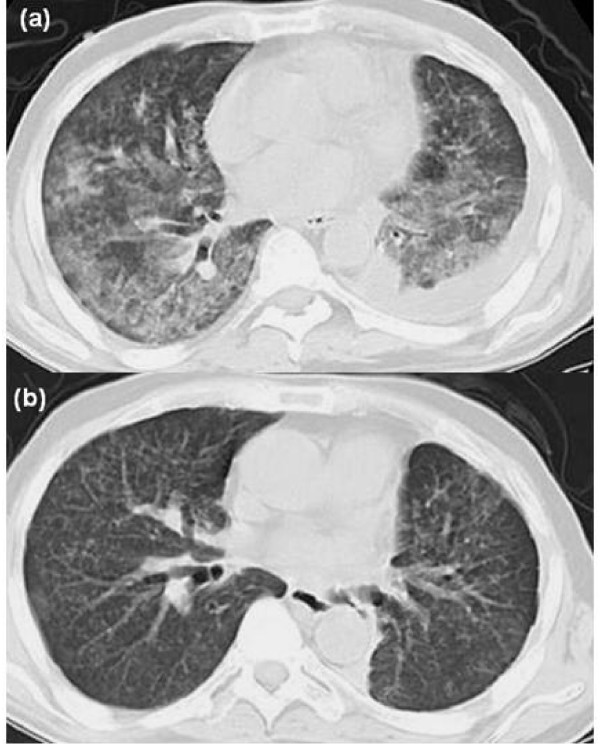
Radiological evaluation of gefitinib-related interstitial lung disease on CT scans. (a) before treatment, (b) after treatment.

However, the patient developed progressive general fatigue and shortness of breath 45 days after the initiation of gefitinib therapy. A chest CT scan demonstrated new areas of patchy ground glass opacity (GGO) accompanied by interstitial markings bilaterally, without evidence of tumor growth (Figure 2a). The serum LDH level was elevated to 457 IU/L (cut-off: 208 IU/L). To rule out infectious etiologies, we performed sputum cultures and relevant stainings for bacteria, fungi, and pneumocystis carinii, and the cytomegalovirous antigen test. None of these examinations were positive. Because of severe respiratory dysfunction, we could not perform bronchoscopy with bronchoalveolar lavage. Cardiogenic etiology was also excluded by electrocardiogram and echo cardiogram. Based on these findings, he was diagnosed as having gefitinib-related ILD, and gefitinib therapy was stopped. He was treated with high-dose corticosteroid (1 g/day of intravenous methylpredonisolone for three days) followed by a maintainace dose of 50 mg/day of oral prednisolone. The dose of oral prednisolone was decreased by 10 mg per week. After one month of steroid therapy, the patient reported marked improvement of dyspnoea. A chest CT scan showed resolution of GGO areas and interstitial markings, however the pulmonary metastatic lesions had slightly grown (Figure 2b). The serum LDH level was normalized two months after the initiation of steroid therapy.

**Figure 3:**
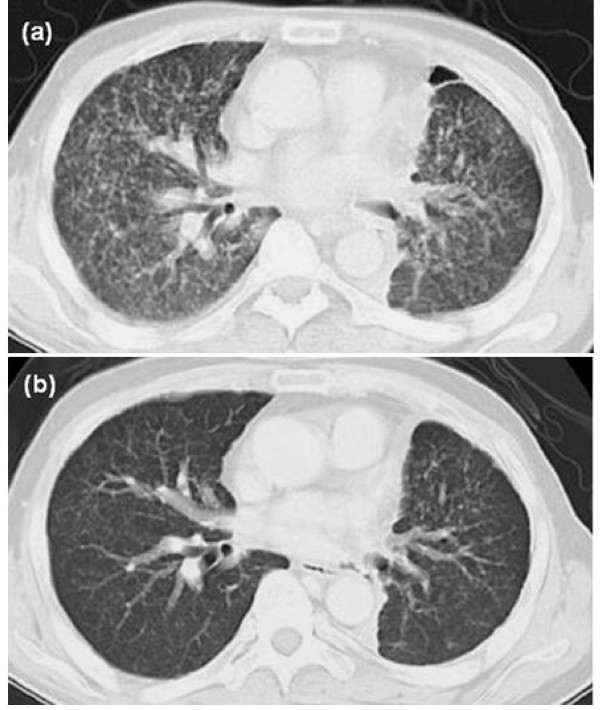
Radiological evaluation of response to re-treatment with gefitinib on CT scans. (a) before re-treatment, (b) after re-treatment.

In the five months following the withdrawal of gefitinib, miliary pulmonary metastases with lymphangitis carcinomatosa gradually progressed (Figure 3a). The patient was confined to bed due to severe dyspnoea and rapidly progressing hypoxia. He was judged to be intolerant to further cytotoxic chemotherapy in consideration of his physical condition. He and his family strongly desired the readministration with gefitinib rather than palliative care. Gefitinib therapy with 50% dose (125 mg/day) was therefore initiated, after receiving informed consent for the use of an unproven treatment dose of gefitinib and the high risk of ILD relapse. Fortunately, the symptoms rapidly improved one week after therapy was resumed. A chest CT scan taken one month later showed a significant response (Figure 3b). Thereafter, disease progression of pulmonary and bone metastases was documented 6 months after readministration of gefitinib. His general condition gradually deteriorated with disease progression, and he expired 16 months after the readministration of gefitinib.

Because he gave his written informed consent to let us use surgically resected specimens for genetic analyses before operation, a mutation analysis of the epidermal growth factor receptor (*EGFR*) gene was conducted at Mitsubishi Kagaku Bio-Clinical Laboratories, Inc. Genomic DNA was prepared from a paraffin-embedded section using macrodissection in a surgically resected primary tumor specimen. The peptide nucleic acid-locked nucleic acid PCR clamp protocol [5] was used to perform a mutation analysis. One of the most common activating mutations, L858R in exon 21, was identified.

## Discussion

The precise management of a gefitinib responder having severe adverse events remains unknown. There are only two case reports on readministration of gefitinib in responders following treatment discontinuation due to severe gefitinib-related hepatotoxicity [6,7]. Although the resumption of gefitinib (250 mg/day) and concurrent steroid therapy [6] failed to control hepatotoxicity, intermittent schedule of gefitinib administration (250 mg/day once every 5 days) [7] not only successfully reduced hepatotoxicity but also induced disease regression.

To the best of our knowledge, this is the second report of readministration of gefitinib in a patient who had once developed gefinitib-related ILD. Yano *et al *[8] previously reported the readministration of gefitinib in a responder after discontinuation owing to ILD(alveolar hemorrhage). Starting with 250 mg/day, gefitinib was given every other day after blepharitis developed at the time of initial treatment. This intermittent schedule of gefitinib administration was effective both for the initial treatment and the resumed treatment.

The currently approved dose of gefitinib was determined based on the data from two large phase II trials (IDEAL 1 and 2). Similar efficacy but higher toxicity was observed with the 500 mg dose. Consequently, 250 mg has been the recommended dose [9,10]. However, anti-tumor activity was not necessarily dependent on the dose of gefitinib in previous trials [1,2,9,10]. Even when the dose of gefitinib was less than 250 mg/day, several responders were documented [1,2]. However, the development of adverse effects other than ILD is usually dependent on the dose of gefitinib [1,2]. Therefore, re-treatment by a reduced dose may be effective for patients who had previously responded to standard dose of gefitinib but had discontinued treatment due to the occurrence of severe adverse events other than ILD. Due to the low incidence of gefinitib-related ILD, it is difficult to show whether the same hypothesis fit to the gefinitib-related ILD based on the observational studies. Although there are no data indicating the dose-dependency of ILD, we could successfully manage a patient who had once developed ILD with a half dose of gefitinib.

Numerous studies suggest that gefitinib has fairly effective anti-tumor activity, especially for tumors with *EGFR *gene mutations [11,12]. It is well-known that *EGFR*-mutated tumors respond dramatically to lower dose of EGFR-TKIs such as gefitinib in experimental studies [13,14]. These findings may suggest that a reduced dose of gefitinib is sufficient for tumors with these molecular characteristics, and that the currently approved dose is excessively potent in some of these patients and results in adverse events.

The prognosis of patients with lymphangitis carcinomatosa is extremely poor, with approximately 50% of the patients dying within three months of their first respiratory symptoms [15]. Fortunately, our patient was able to spend the rest of his life at home without the relapse of ILD for 16 months after readministration of gefitinib. Therefore, a resumption of gefitinib therapy would be clinically beneficial for him. However, secondary treatment with gefitinib for a patient who once developed gefinitib-related ILD is usually considered very risky. Therefore, at this time, alternative therapeutic modalities should be chosen, if available.

## Conclusion

We herein presented the case of gefitinib readministration in a patient with lung adenocarcinoma who had once responded but in whom treatment had to be discontinued owing to gefinitib-related ILD. Gefitinib readministration with 50% dose was found to successfully control disease progression.

We believe clinical trials are warranted to evaluate gefitinib readministration with a dose reduction for patients who have once responded but later discontinued this treatment owing to severe adverse events including ILD. The appropriate dose and schedule of gefitinib readministration also should be determined in these trials.

## Abbreviations

CT: computed tomography; 

EGFR: epidermal growth factor receptor

GGO: ground glass opacity;

ILD: interstitial lung disease;

WJTOG: West Japan Thoracic Oncology Group; 

## Competing interests

The author(s) declare that they have no competing interests.

## Authors' contributions

**KT **reviewed the medical records and imaging findings, drafted the manuscript and coordinated the submission. KF ans KS participated in care of the patient, and helped draft the manuscript. AHB critically revised the manuscript. KY, TM, and TI participated in reviewing the manuscript. All authors read and approved the final manuscript.

## Consent

Written informed consent was given for publication from the patient's next-of-kin.

## References

[B1] Ranson M Hammond LA Ferry D Kris M Tullo A Murray PI Miller V Averbuch S Ochs J Morris C ZD a selective oral epidermal growth factor receptor-tyrosine kinase inhibitor, is well tolerated and active in patients with solid, malignant tumors: results of a phase I trial J Clin Oncol 1839 20 2240 50 10.1200/JCO.2002.10.112 11980995

[B2] Baselga J Rischin D Ranson M Calvert H Raymond E Kieback DG Kaye SB Gianni L Harris A Bjork T Phase I safety, pharmacokinetic, and pharmacodynamic trial of ZD a selective oral epidermal growth factor receptor tyrosine kinase inhibitor, in patients with five selected solid tumor types J Clin Oncol 1839 20 4292 302 10.1200/JCO.2002.03.100 12409327

[B3] Inoue A Saijo Y Maemondo M Gomi K Tokue Y Kimura Y Ebina M Kikuchi T Moriya T Nukiwa T Severe acute interstitial pneumonia and gefitinib Lancet 2003 361 137 9 10.1016/S0140-6736(03)12190-3 12531582

[B4] Ando M Okamoto I Yamamoto N Takeda K Tamura K Seto T Ariyoshi Y Fukuoka M Predictive factors for interstitial lung disease, antitumor response, and survival in non-small-cell lung cancer patients treated with gefitinib J Clin Oncol 2006 24 2549 56 10.1200/JCO.2005.04.9866 16735708

[B5] Nagai Y Miyazawa H Huqun Tanaka T Udagawa K Kato M Fukuyama S Yokote A Kobayashi K Kanazawa M Genetic heterogeneity of the epidermal growth factor receptor in non-small cell lung cancer cell lines revealed by a rapid and sensitive detection system, the peptide nucleic acid-locked nucleic acid PCR clamp Cancer Res 2005 65 7276 82 10.1158/0008-5472.CAN-05-0331 16105816

[B6] Ho C Davis J Anderson F Bebb G Murray N Side effects related to cancer treatment: CASE 1. Hepatitis following treatment with gefitinib J Clin Oncol 2005 23 8531 3 10.1200/JCO.2004.00.6650 16293881

[B7] Seki N Uematsu K Shibakuki R Eguchi K Promising new treatment schedule for gefitinib responders after severe hepatotoxicity with daily administration J Clin Oncol 2006 24 3213 4 author reply 3214-5. 10.1200/JCO.2006.05.7109 16809744

[B8] Yano S Nakataki E Ohtsuka S Inayama M Tomimoto H Edakuni N Kakiuchi S Nishikubo N Muguruma H Sone S Retreatment of lung adenocarcinoma patients with gefitinib who had experienced favorable results from their initial treatment with this selective epidermal growth factor receptor inhibitor: a report of three cases Oncol Res 2005 15 107 11 16119008

[B9] Fukuoka M Yano S Giaccone G Tamura T Nakagawa K Douillard JY Nishiwaki Y Vansteenkiste J Kudoh S Rischin D Multi-institutional randomized phase II trial of gefitinib for previously treated patients with advanced non-small-cell lung cancer (The IDEAL 1 Trial) [corrected] J Clin Oncol 2003 21 2237 46 10.1200/JCO.2003.10.038 12748244

[B10] Kris MG Natale RB Herbst RS Lynch TJ Jr Prager D Belani CP Schiller JH Kelly K Spiridonidis H Sandler A Efficacy of gefitinib, an inhibitor of the epidermal growth factor receptor tyrosine kinase, in symptomatic patients with non-small cell lung cancer: a randomized trial Jama 2003 290 2149 58 10.1001/jama.290.16.2149 14570950

[B11] Mitsudomi T Kosaka T Endoh H Horio Y Hida T Mori S Hatooka S Shinoda M Takahashi T Yatabe Y Mutations of the epidermal growth factor receptor gene predict prolonged survival after gefitinib treatment in patients with non-small-cell lung cancer with postoperative recurrence J Clin Oncol 2005 23 2513 20 10.1200/JCO.2005.00.992 15738541

[B12] Han SW Kim TY Hwang PG Jeong S Kim J Choi IS Oh DY Kim JH Kim DW Chung DH Predictive and prognostic impact of epidermal growth factor receptor mutation in non-small-cell lung cancer patients treated with gefitinib J Clin Oncol 2005 23 2493 501 10.1200/JCO.2005.01.388 15710947

[B13] Lynch TJ Bell DW Sordella R Gurubhagavatula S Okimoto RA Brannigan BW Harris PL Haserlat SM Supko JG Haluska FG Activating mutations in the epidermal growth factor receptor underlying responsiveness of non-small-cell lung cancer to gefitinib N Engl J Med 2004 350 2129 39 10.1056/NEJMoa040938 15118073

[B14] Paez JG Janne PA Lee JC Tracy S Greulich H Gabriel S Herman P Kaye FJ Lindeman N Boggon TJ EGFR mutations in lung cancer: correlation with clinical response to gefitinib therapy Science 2004 304 1497 500 10.1126/science.1099314 15118125

[B15] Bruce DM Heys SD Eremin O Lymphangitis carcinomatosa: a literature review J R Coll Surg Edinb 1996 41 7 13 8930034

